# Estimation of Soil Depth Using Bayesian Maximum Entropy Method

**DOI:** 10.3390/e21010069

**Published:** 2019-01-15

**Authors:** Kuo-Wei Liao, Jia-Jun Guo, Jen-Chen Fan, Chien Lin Huang, Shao-Hua Chang

**Affiliations:** 1 Department of Bioenvironmental Systems Engineering, National Taiwan University, No. 1, Section 4, Roosevelt Rd., Taipei 10617, Taiwan; 2 Hetengtech Company Limited, New Taipei City 24250, Taiwan

**Keywords:** slopeland, Bayesian Maximum Entropy, soil depth, physiographic factors

## Abstract

Soil depth plays an important role in landslide disaster prevention and is a key factor in slopeland development and management. Existing soil depth maps are outdated and incomplete in Taiwan. There is a need to improve the accuracy of the map. The Kriging method, one of the most frequently adopted estimation approaches for soil depth, has room for accuracy improvements. An appropriate soil depth estimation method is proposed, in which soil depth is estimated using Bayesian Maximum Entropy method (BME) considering space distribution of measured soil depth and impact of physiographic factors. BME divides analysis data into groups of deterministic and probabilistic data. The deterministic part are soil depth measurements in a given area and the probabilistic part contains soil depth estimated by a machine learning-based soil depth estimation model based on physiographic factors including slope, aspect, profile curvature, plan curvature, and topographic wetness index. Accuracy of estimates calculated by soil depth grading, very shallow (<20 cm), shallow (20–50 cm), deep (50–90 cm), and very deep (>90 cm), suggests that BME is superior to the Kriging method with estimation accuracy up to 82.94%. The soil depth distribution map of Hsinchu, Taiwan made by BME with a soil depth error of ±5.62 cm provides a promising outcome which is useful in future applications, especially for locations without soil depth data.

## 1. Introduction

Soil depth plays an important role in landslide disaster prevention and management. Soil depth is an important parameter in deterministic analysis for shallow landslide prediction and landslide mass estimation. In Taiwan, soil depth is also used as for slopeland control by local governments. The Taiwan Slopeland Conservation and Utilization Act, 2016 defines soil depth as the distance from land surface to a specified depth, which confines roots of plants from further growth and grades them in four levels, as shown in Table 1. The grade of a slopeland is a key factor in determining whether it is applicable for agriculture or forestation only. Because this grading system is widely used by engineers, it is adopted here as the soil category system.

There are two sources of soil depth information available now: the slopeland soil depth distribution maps by the Soil and Water Conservation Bureau, Council of Agriculture, Executive Yuan in 1995. These maps suffer not only from being outdated data with low accuracy and poor space estimation, but also from limited coverage. The other available soil depth source is the manually measured soil depth by the Taiwan Agricultural Research Institute, Council of Agriculture, Executive Yuan. In spite of its high accuracy, the measured soil depths also suffer from the same drawback of limited coverage. As soil depth is usually in close correlation with topography [1,2], many scholars have managed to estimate soil depth by the Digital Elevation Model (DEM), a 3D digital representation of ground surface topography or terrain, and geostatistical methods.

Soil depth estimation relied on the Kriging method in the past. For example, Bourennane et al. [3] estimated soil depth with the Kriging method aligned with linear regression and ended up with more accurate estimates of soil depth than the Kriging method alone. Kuriakose et al. [4] employed regression in the Kriging method to estimate soil depth in a smaller area (9.5 square kilometers in Western Ghats of Kerala, India). He divided the site into 20 m × 20 m cells and executed the estimation based on environment variables including elevation, slope, aspect, curvature, topographic humidity index, use of land, and distance between sample points and rivers. Compared with other methods, the Kriging method appears ideal in predicting the spatial distribution of soil depth.

Most traditional geostatistical methods not only have to rely on normal distribution and linear estimation, but are also limited to analysis and study on hard data by actual measurement. This results in some new time-space geostatistical methods, e.g., the Bayesian Maximum Entropy (BME method). BME is not limited by Gaussian distribution and linear estimation, and combines physics knowledge or other probabilistic (soft) data with the concept of Bayesian conditional probability to gradually enhance estimation information and estimate stochastic and spatial data [5,6,7,8,9,10] at the same time. Since its introduction in 1991, BME has been widely used in various fields of science and engineering and proven to be more accurate than traditional spatial statistical methods [7,11,12].

Focused on the Hsinchu district in Taiwan, this study estimates soil depth based on hard and soft soil depth data using BME. For the first time, the BME is used to estimate the soil depth. According to Taiwan’s classification of soil depth, the estimated soil depth is then divided into four levels: shallow layer (<20 cm), shallow layer (20–50 cm), deep layer (50–90 cm), and deep layer (>90 cm). This study also compares the accuracy of the results of the traditional Kriging method against the soil depth maps of Taiwan with the goal of identifying a valid space distribution estimation method to provide soil depth data in Taiwan. 

## 2. Research Material and Methodology 

### 2.1. Research Material 

#### 2.1.1. Research District

**Figure 1:**
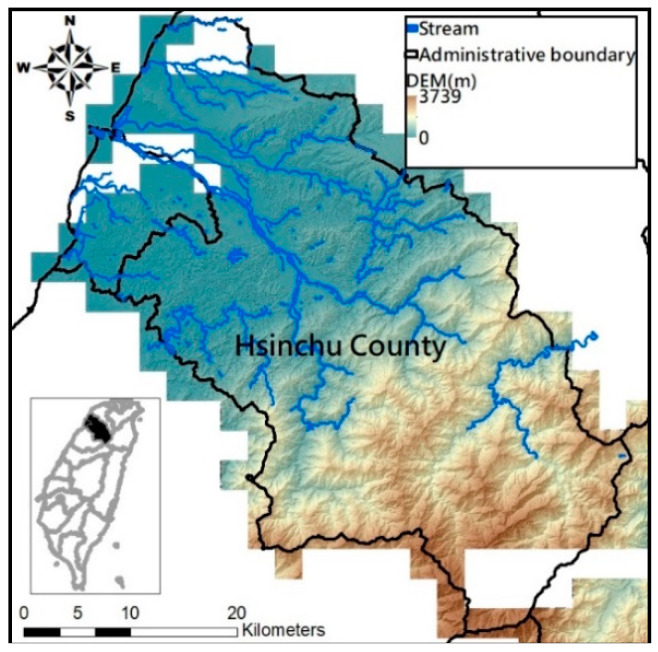
Elevation distribution and administrative division of Hsinchu district.

This study takes the Hsinchu district in Taiwan for research. Located in the northwestern part of Taiwan island, Hsinchu city and county span 1532 square kilometers east of the Taiwan Strait. Most of the district is occupied by hills, terraces, and mountains and with population concentrated in the alluvial plains in the lower reaches of the river. Its annual average temperature is about 22.6 degrees, rainfall about 1718.1 mm in 115.5 days, and with rainy season from May to September. The elevation distribution and geographical location of Hsinchu area are shown in Figure 1.

#### 2.1.2. Soil Depth

**Figure 2:**
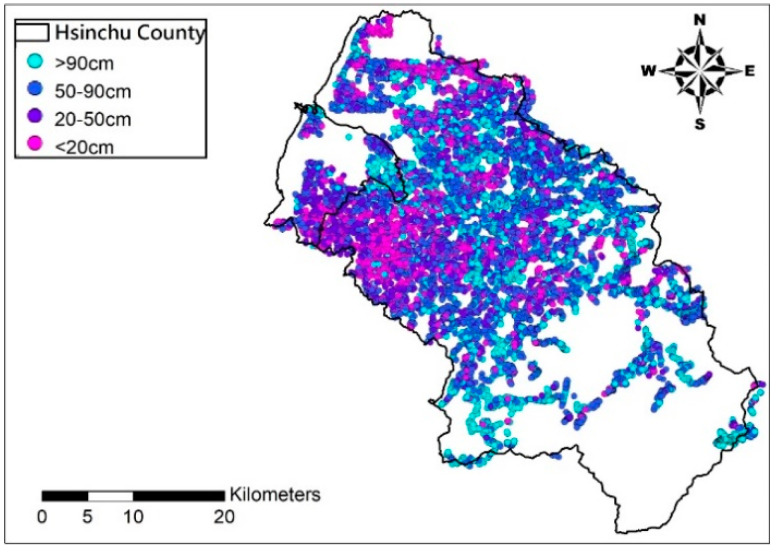
Locations of measured soil depth in Hsinchu County.

**Figure 3:**
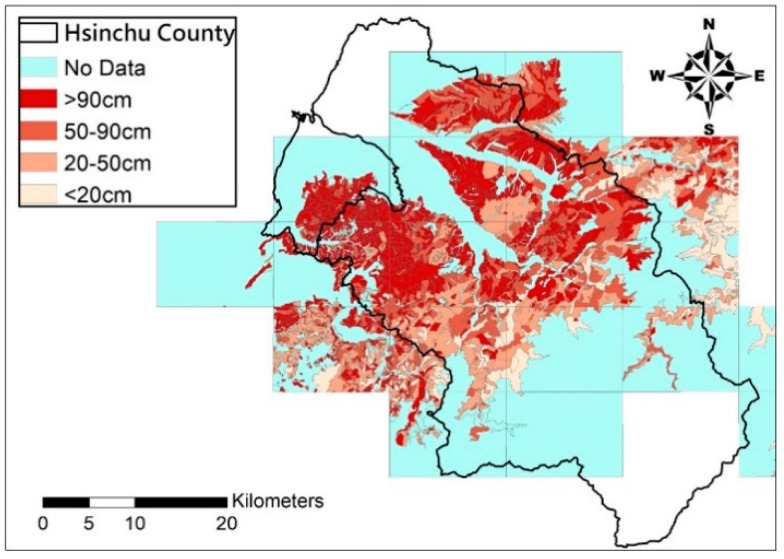
Current soil depth map for Hsinchu County.

This study uses 8217 soil depth records by the Agricultural Research Institute from drilling in this district in 2010, 2011, and 2016, as the basic data for the estimation model; the slopeland soil depth distribution maps by the Soil and Water Conservation Bureau in 1995 are taken as the control group. The soil drilling points and soil depth maps are shown in Figure 2 and Figure 3, respectively.

### 2.2. Research Methodology 

#### 2.2.1. Kriging Estimation

The Kriging estimation method develops system equations based on characteristics possessed by regionalized variables to estimate position of any point in the space with known regionalized variable *Z*$\left(x_{i}\right)$ (*i* = 1, 2, …, *n*) in a random field. For any position with a known relative distance, the Kriging method may be used to estimates its Z^*^$\left(x_{0}\right)$. The method first presents the distance function of soil depth measurement (point data) in the semivariogram model and determines the weight coefficient of each point $\left(\lambda_{0i}\right)$ with unbiased optimization from semivariogram. The semivariogram measures things nearby tend to be more similar than things that are farther apart, revealing the strength of statistical correlation as a function of distance. The soil depth then can be estimated using a linear combination of the weight coefficient and the measured data. The semivariance (*γ*) can be calculated by Equation (1), where *h* is distance between two separated points, *N*(*h*) is the set of all pairwise Euclidean distances *i* − *j* = *h*, |*N*(*h*)| is the number of pairs in *N*(*h*).

(1) $$\gamma \left(h\right)=\frac{\sum_{N(h)}{\left[z(x_{i})-z(x_{j})\right]}^{2}}{2\left|N(h)\right|}$$

The difference between the simple Kriging (SK) and the ordinary Kriging (OK) is the assumption of stationarity. SK utilizes this assumption while OK does not. The focus of this research is to utilize BME for estimating the soil depth. Thus, whether or not the stationarity has an effect on the accuracy of the Kriging model is not further discussed. Thus, only OK with three different semivariogram models were investigated. The OK used in this study for soil depth estimation is executed in steps described below:

i. Calculate the semivariance and distance between two measured points (ranging from meters to kilometers), calculate the average of semivariance and distance of points in the district as the representative value of the district, connect the latter to obtain the experimental semivariogram, overlay it with commonly used theoretical semivariogram models (index, spherical, and Gaussian).
ii. Organize and calculate characteristics of the Best Linear Unbiased Estimator (BLUE) possessed by the ordinary Kriging method. The term of “best” indicates that compared to other unbiased and linear estimators, the lowest variance of the estimate is given
iii. Acquire data *Z*_1_ of one point out of n-each soil depth measurements, estimate *Z*_1_ with Kriging method based on the remaining *n* − 1 points. Here, *n* (i.e., 8217) is the data points used in the Kriging model.
iv. Replace *Z*_1_ with another point *Z*_2_ and repeat the same steps until all of the *n*-each points are estimated.
v. Compare the soil depth measurements and Kriging method estimates according to the four soil depth gradings.
vi. Calculate the average estimation accuracy of the index, spherical, and Gaussian theoretical semivariogram models in each watershed.

#### 2.2.2. Bayesian Maximum Entropy Method (BME)

BME divides knowledge into two categories: General Knowledge Base (G-KB), containing time-space related statistical knowledge such as mean, covariance, and semivariation and the site-specific knowledge base (S-KB), containing hard and soft data types. The hard data type are actual measurements and exact values, while the soft ones are presented by a probability density function (PDF). The entire knowledge base, the comprehensive knowledge G-KB and the specific point knowledge S-KB combined, can be expressed as K = G ∪ S.

BME generally consists of three epistemological stages: a prior stage, a pre-posterior (or meta-prior) stage, and a posterior stage [7,13]. In the prior stage, the joint PDF *f*_G_(*x*_map_), given general knowledge *G*, is calculated via the maximum entropy theory. The variable *x*_map_ is a vector of points, *x*_soft_, *x*_hard_, and *x_k_*, representing the information of the soft and hard data points and unknown values at the estimation point, respectively. The expected information is expressed as Equation (2):

(2) $$\bar{{\mathrm{Info}}_{G}[x_{\mathrm{map}}]}=-\int dx_{\mathrm{map}}f_{G}(x_{\mathrm{map}})\log f_{G}(x_{\mathrm{map}})$$

The general knowledge *G* in Equation (14) is expressed as *g_α_*(*x*_map_), a set of functions of *x*_map_ such as the mean and covariance moments. To obtain the prior PDF of *f_G_*(*x*_map_), the expectation of Equation (2) is maximized with consideration of *g_α_*(*x*_map_). Equation (3) is the object function if the Lagrange multipliers method (LMM) is adopted for the aforementioned maximization problem, in which *μ_α_* is the Lagrange multiplier and the *E*[*g_α_*(*x*_map_)] is the expected value of *g_α_*(*x*_map_).

(3) $$\begin{array}{ll} \mathrm{Max}.\;[f_{G}(x_{\mathrm{map}})] & =-\int dx_{\mathrm{map}}f_{G}(x_{\mathrm{map}})\log f_{G}(x_{\mathrm{map}})\\ & -\sum_{\alpha }^{N}\mu_{\alpha }\left[\int g_{\alpha }f_{G}(x_{\mathrm{map}})f_{G}(x_{\mathrm{map}})dx_{\mathrm{map}}-E[g_{\alpha }(x_{\mathrm{map}})]\right] \end{array}$$

At the pre-prior stage, new information, which can be hard data or soft data, is collected for the points to be estimated. The hard data could be actual measurements and the soft data could be in various forms but is not used at the prior stage [7]. At the posterior stage, the posterior PDF *f_K_*(*x_k_*|*x*_data_) is derived using Bayesian theory, resulting in the following equations:

(4) $$f_{K}(x_{k}|x_{data})=A^{-1}\int dx_{\mathrm{soft}}f_{S}(x_{\mathrm{soft}})f_{G}(x_{\mathrm{map}})$$

(5) $$A=\int dx_{\mathrm{soft}}f_{S}(x_{\mathrm{soft}})f_{G}(x_{\mathrm{data}})$$

in which *x*_data_ is a pointer for a context of knowledge, and (*x_k_*|*x*_data_) stands for the possible values *x_k_* of the map in the context specified by *x*_data_. In this study, the soil depth measurements and the physiographic factor out of 5 m DEM are source of estimates information which can be expressed in formula $S:X_{\mathrm{data}}=\left(X_{\mathrm{hard}},X_{\mathrm{soft}}\right)=\left(x_{1},\ldots ,x_{n}\right)$ according to information classification by S-KB; here $X_{\mathrm{hard}}=\left(x_{1},\ldots ,x_{m_{h}}\right)$ is the soil depth measurement of $P_{i}\left(i=1,2,\ldots ,m_{h}\right)$; $X_{soft}=\left(x_{m_{h}+1},\ldots ,x_{n}\right)$ is the soil depth estimates on point $P_{i}\left(i=m_{h}+1,\ldots ,n\right)$. by the soil depth estimation model and soil depth relevant physiographic factor. The soil depth can be regarded as a space random field with the soil depth of any point in the field expressed by formula $X_{P}=X_{s}$ with p=(s) where *s* is the space coordinate. This study takes distribution characteristics of soil depth in space into account and express soil depth of every point in space with $f_{KB}$, the PDF; where KB is the knowledge base (KB) used when constructing this PDF. 

(1) Operation process

The input data of BME may contain hard data and soft data. The hard ones are soil depth measurements, while the soft ones are soil depth estimated from the built prediction model, such as Kriging with input of the soil depth measurement and their relevant physiographic factor on the DEM. After all estimates are included, set soft data to low frequency (data trend) and deduct it from the hard data to obtain high frequency (data residual); consider the covariance distribution of data residual as the input of the BME method to estimate soil depth residual difference of unknown points; combine the latter with the data trend established earlier to build the final distribution of soil depth in space.

(2) Soft data

This study employs the soil-drilling data of the Hsinchu district provided by the Agricultural Research Institute and relevant physiographic factors geologic factors as the input data for estimating effective soil depth and create low frequency data trend with AI model LSSVM (Least Squares Support Vector Machine) and the nonlinear model SVR (Support Vector Regression). The physiographic factors used include slope, aspect, profile curvature, plan curvature, and topographic wetness index. This study employs 80% of the soil drilling data to train the model before using it to estimate soil depth of the remaining 20% of the drilling points, and compares the estimates and actual soil depth measurements to assess both methods (LSSVM and SVR) to build up the estimation model for soil depth soft data.

i. Pparation of Physiographic Factor

This study employs the DEM at 5 m resolution along with the physiographic factor adopted by Kuriakose et al. [4] to create physiographic factors by GIS software ArcGIS10.1, including slope, aspect, profile curvature, plan curvature, and topographic wetness index (TWI), in the Hsinchu district for soil depth estimation in future. Note that TWI is a function of both the slope and the upstream contributing area per unit width orthogonal to the flow direction. Table 2 displays the outcome of and correlation between physiographic factor preparation.

ii. LSSVM

Support Vector Machine (SVM) is a method for classification or regression. That is, one can train an SVM with a group of classified data and use it to estimate type of a piece of data in an unclassified group. Quadratic Programming (QP) usually adopted in solving the above optimization problem suffers from complex calculation as the restriction of SVM is an inequality. The LSSVM is an improved version of SVM with simpler and faster calculation. LSSVM was developed by Suykens, et al. [14] who introduced the concept of Least Squares Loss Function in SVM. A standard SVM, as described in Equation (6), solves a nonlinear classification problem by means of convex quadratic programs (QP).

(6) $$\begin{array}{l} \underset{w,b,\xi }{\mathrm{minimize}}\;\frac{1}{2}w^{T}w+c\sum_{k=1}^{N}\xi_{k}\\ \mathrm{Subject}\;\mathrm{to}\;\{\begin{array}{c} y_{k}(w^{T}K(x_{i})+b)\geq 1-\xi_{k}\\ \xi_{k}\geq 0,\;i=1,2,\ldots ,N \end{array} \end{array}$$

where *w* is a normal vector to the hyper-plane; *c* is a real positive constant; and $\xi_{k}$ is the slack variable. If $\xi_{k}$ > 1, the *k*-th inequality becomes violated compared to the inequality from the linearly separable case. *y_k_* is the class; [*w^T^K*(*x_i_*) *+ b*] is the classifier; *N* is the number of data; and *K* is the kernel function. In the current study, the Gaussian radial basis function (RBF) kernel is used, as shown in Equation (7):

(7) $$K(X,X_{i})=e^{-\sigma {\left(||X-X_{i}||\right)}^{2}}$$

where *X* is the input vector, *σ* is the kernel function parameter; and *X_i_* are the support vectors. LSSVM [14], instead of solving the QP problem, solves a set of linear equations by modifying the standard SVM, as described in Equation (8):

(8) $$\begin{array}{l} \min\;\frac{1}{2}w^{T}w+\frac{\gamma }{2}\sum_{k=1}^{N}e_{k}^{2}\\ s.t.\;y_{k}(w\cdot K(x_{k})+b)=1-e_{k},\;k=1,\ldots ,n \end{array}$$

where *γ* is a constant number and *e* is the error variable. Compared to the standard SVM, there are two modifications leading to solving a set of linear equations. First, instead of inequality constraints, the LSSVM uses equality constraints. Second, the error variable is a squared loss function.

iii. SVR

SVR differs from SVM in that SVM aims to find a plane to divide the data into two, while SVR is a plane accurately predicting data distribution. The main concept is to give a fault tolerance upper limit to execute regression analysis and leave a deterministic range of fault tolerance for errors of each data point. This prevents the regression model from over-fitting. With a similar mathematical model of SVM, the linear model of SVR may be converted into non-linear one with the kernel trick, which, in turn, can process more complex data with even better prediction results.

iv. Model establishment and evaluation

The accuracy of these two soil depth estimation models (LSSVM and SVR) is determined by comparing actual soil depth data. Acquire 80% of physiographic factor and soil depth data of the Hsinchu district to train both models, the remaining 20% soil depth data is used to assess accuracy of models, in which 10-fold cross validation is adopted.

In addition to the LSSVM and SVR models, the K Nearest Neighbor (KNN) algorithm is also considered. KNN estimates the target soil depth by averaging 10 nearest soil depth values. This study employs three indices, *R*^2^, MAPE, and hitting rate of the current soil depth grading, to assess accuracy of estimation by each model. An estimate is defined as “targeted” if both estimate and actual soil depth fall in the same soil depth grade. The formulae for the three indices are shown below:

(9) $$R^{2}＝1-\frac{\sum^{\text{​}}{\left(y_{i}-\hat{y}\right)}^{2}}{\sum^{\text{​}}{\left(y_{i}-\bar{y}\right)}^{2}}$$

(10) $$MAPE=\frac{\sum^{\text{​}}\left|y_{i}-\hat{y}\right|}{y_{i}}\times \frac{100}{N}\;(\%)$$

(11) $$\mathrm{Hitting}\;\mathrm{rate}\left(\%\right)=\frac{m}{N}$$

where $y_{i}$ is the actual soil depth, $\hat{y}$ is the soil depth estimates, $\bar{y}$ is the average actual soil depth, *N* is the number of data entries, and *m* is the number of actual and estimate soil depth pairs that fall in the same grade. 

(3) Nested spatiotemporal covariance model

This study correlates residual data with the covariance model to estimate the high frequency data. The covariance model $C_{st}\left(h,\tau \right)$ is shown in Equation (12).

(12) $$C_{st}\left(h,\tau \right)=\overset{N}{\underset{l=1}{\sum }}b_{l}C_{sl}\left(h;A_{sl}\right)C_{tl}\left(\tau ;A_{tl}\right)$$

where *s* is space, *t* is time, *h* is spatial distance, *τ* is time distance, *N* is nest count, $b_{l}$ denotes threshold value (sill), and $C_{sl}\left(h;A_{sl}\right)$ is a space covariance model alone, $C_{tl}\left(\tau ;A_{tl}\right)$ is a time covariance model alone. Gaussian and Exponential are two commonly available patterns for covariance model, $A_{sl}$ and $A_{tl}$ are parameters required by $C_{sl}$ and $C_{tl}$, i.e., the range of variances.

The nested covariance model is then used to determine model-data fitness based on the Akaike information criterion (AIC) as shown in Equation (13).

(13) $$AIC=2k+nln\left(\frac{RSS}{n}\right)$$

where *k* is the number of parameters used, *n* is the number of measurements, and *RSS* is the residual sum of squares, and the smaller the AIC, the closer to the target. 

#### 2.2.3. Method of Accuracy Calculation

This study employs a confusion matrix to assess the accuracy of the Kriging method and BME. The estimates and actual measurement data are classified before inserting their counts in the confusion matrix as indicated in Table 3; soil depth grades are shown in rows of the confusion matrix, while actual soil depth measurement is shown in the columns. For example, in case soil depth estimation of a point is “very shallow” while the actual soil depth in grade “shallow” then insert it in cell (2) of Table 3 and counts this prediction to “invalid estimation”. For another point with correct estimation for grade “deep”, then add the value in cell (14) of Table 3, that is, this prediction is considered as a “valid estimation”. Table 3 suggests the number of valid estimation points is in cell (1), (6), (11), and (16) while all the remaining cells are invalid estimations. If the total number of points is *n*, then the accuracy rate is expressed by Equation (14):

(14) $$\mathrm{Accuracy}\;\mathrm{rate}=\frac{n_{\left(1\right)}+n_{\left(6\right)}+n_{\left(11\right)}+n_{\left(16\right)}}{n}\times 100\%$$

## 3. Results and Discussion

### 3.1. Existing Soil Map in Hsinchu District

The accuracy of the current slopeland soil map, provided by the Soil and Water Conservation Bureau, is estimated by comparing the measured results of soil drilling. The number of soil drilling measurements is 8217, while only 4768 points are available on slopeland soil map. Table 4 suggests only 1354 points have the same grades by measurement and mapping, i.e., the accuracy rate of the map is only 28.4%. Two causes may have contributed to this low rate. One is that the scale of slopeland soil map is 1/25,000 which is too coarse to reflect space distribution of soil depth; the other is that the map is not up-to-date and failed to consider the impact of relevant physiographic factors on soil depth. Note that although the total data number used here are different from those of Kriging and BME, the average accurate rate is used here and this should be a fair comparison since, for any case, the data number used is more than 4000 points. As shown in Figure 2, it is seen that points categorized into the same grade level often possess a closer distance. That is, although the comparison is performed within the same category, the distance effect is implicitly considered.

### 3.2. Kriging Method

**Figure 4:**
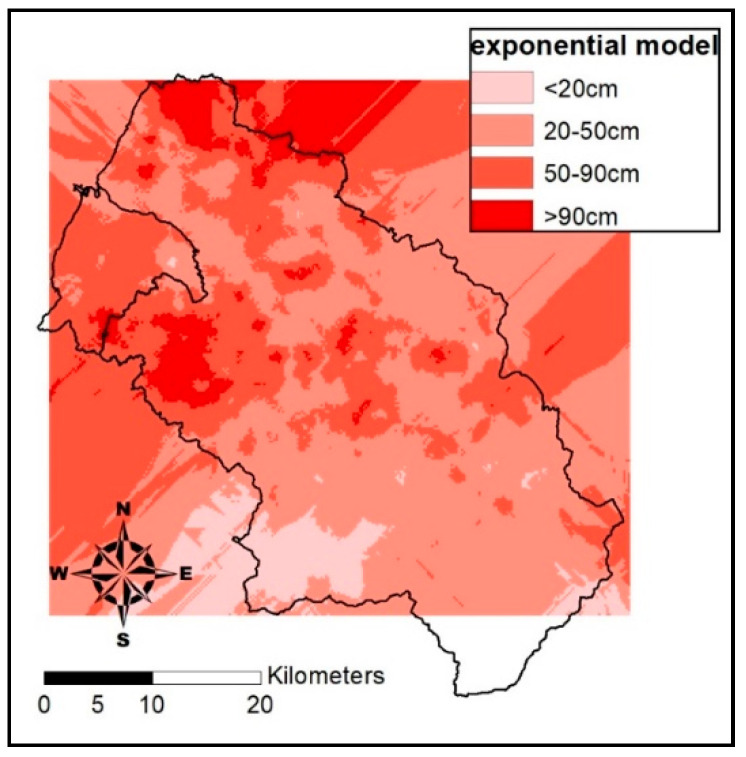
Soil depth estimates by the exponential Kriging model.

**Figure 5:**
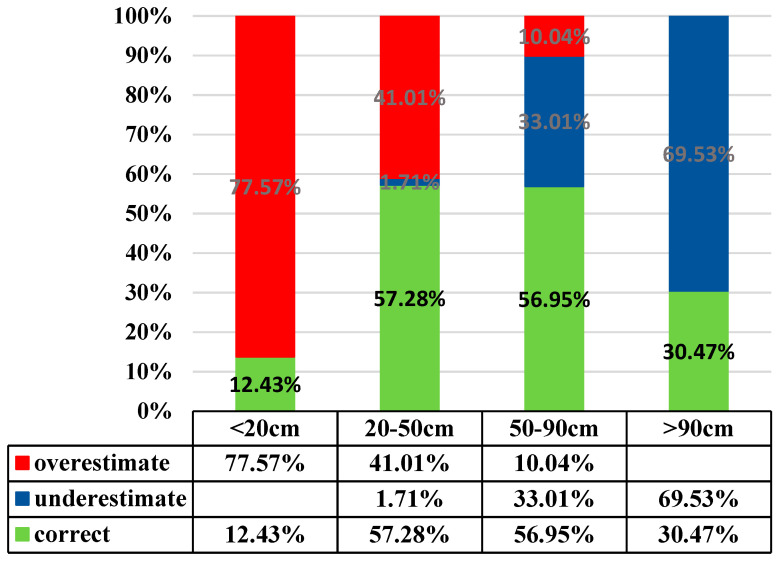
Estimates at different depths by the exponential Kriging model.

**Figure 6:**
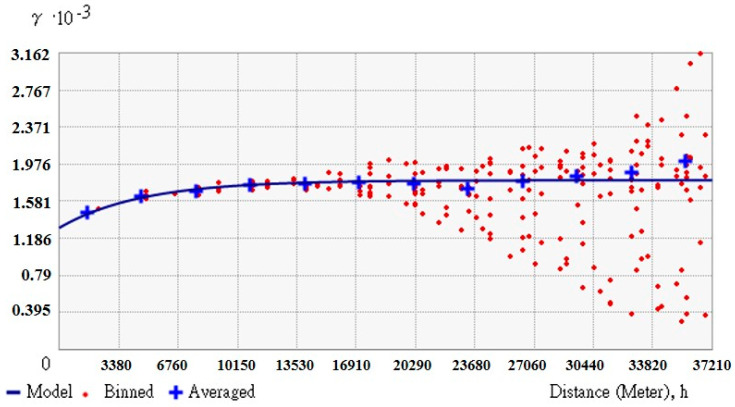
Analysis results of the exponential Kriging model semi-variogram.

Table 5 displays estimates of the three semivariogram models, Exponential model, Spherical, and Gaussian. All have similar accuracy rates, around 40%, with the exponential Kriging method being a little better at 40.4%. Figure 4 displays soil depth estimates by the exponential Kriging model. Detailed results of the exponential Kriging model are displayed in Figure 5, suggesting that overshoot or undershoot are more likely to happen in the “very shallow” and “very deep” grades than in grade “shallow” and “deep”. The accuracy rate may be much higher, at 56.95% and 57.28%, respectively. The estimation of soil depth scores was better for grades “shallow” and “deep” and worse for “very shallow” and “very deep”. The poorer estimates may have been caused by the estimation theory employed by the Kriging method. It is based on the characteristics of distribution of data in space without considering the impact of relevant physiographic factors. The semivariogram model-based estimates shown in Figure 6 do not indicate significant relation between soil depth data and space distribution, i.e., increasing distance does not lead to significant changes in semi-variance. Note that in Figure 6, the values of sill and nugget are 3.162 × 10^−^^3^ and 1.186 × 10^−^^3^, respectively.

### 3.3. BME

#### 3.3.1. Soft Data and Covariance Model

Table 6 shows the results of $R^{2}$, mean absolute percentage error (MAPE), and estimated hitting rate by the K Nearest Neighbor (KNN) algorithm, LSSVM, nonlinear mode SVR, and their combinations. In spite of having a better hitting rate, the MAPE values of KNN suffer larger differences between measured and estimated data. Thus, the KNN results are considered as a factor in LSSVM and SVR, that is, the LSSVM+KNN and SVR+KNN models. As indicated in Table 6, the SVR+KNN model delivers the best hitting rate and least MAPE value. Thus, SVR+KNN is selected as the machine learning model for low frequency data in the proposed BME approach. That is, the outcomes of SVR+KNN is one of the inputs of the proposed BME.

**Figure 7:**
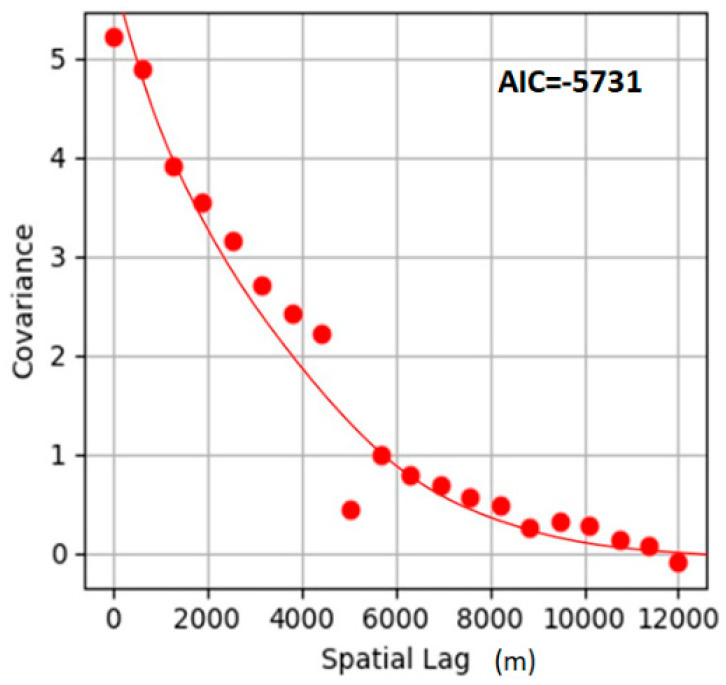
Covariance of residual discrepancy.

The residual covariance function used in this study is displayed in Equation (12), with its distribution shown in Figure 7. The proposed covariance function has one nest, exponential type, and is a space model. As indicated in Figure 7, the high-frequency data (soil depth residual) derived from hard data and soft data have obvious correlation in spatial distribution. The closer the points, the larger the covariance. The covariance may approach zero when the distance between points is extended to 12,000 m^2^. Equation (15) is a good expression of the characteristics of soil depth residual in spatial distribution as its AIC value is −5731.

(15) $$C_{S}\left(h\right)=6.1\mathrm{ExpC}\left(h,9808.84\right)$$

#### 3.3.2. Estimates

**Figure 8:**
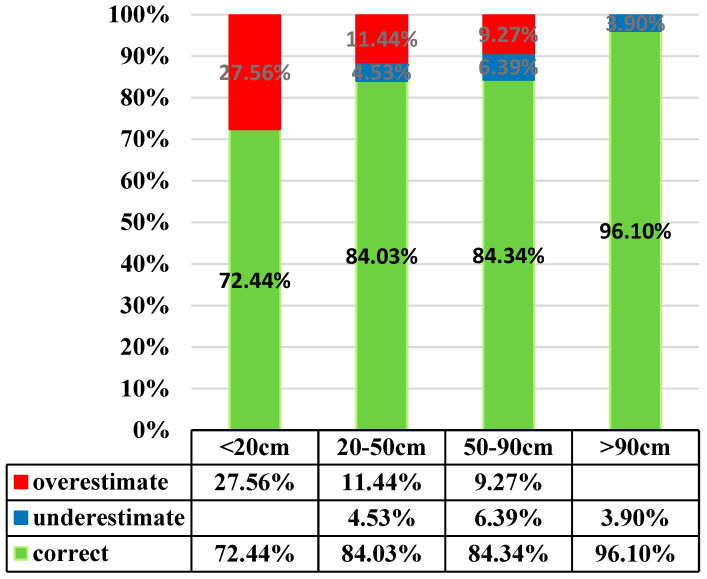
Estimates at different depths by the BME model.

Table 7 displays the accuracy rate of BME estimation. As shown, BME delivers a promising estimation with 82.94% accuracy rate. Differences between soil depth by actual measurement and estimation are two or fewer grades apart. There is no case where the estimates fall in the range of very shallow or very deep while their actual measurements fall in the other opposite grades. Cases of “two-grade” differences are few, 19 out of 8217. The BME method tends to overshoot as there are 1114 positions that suffer higher estimation, while only 288 ones end up with lower estimation. Most overshoot estimates are in the very shallow grade and the undershoot is in shallow grade. This is not the case in terms of percentage: rate of undershooting is highest in the deep grade. As shown in Figure 8, estimates for soil depth in very shallow grade has the most error, shallow and deep grade are about the same accuracy rate, and the very deep grade has makes the fewest error. That is, deeper soils give better estimates.

**Figure 9:**
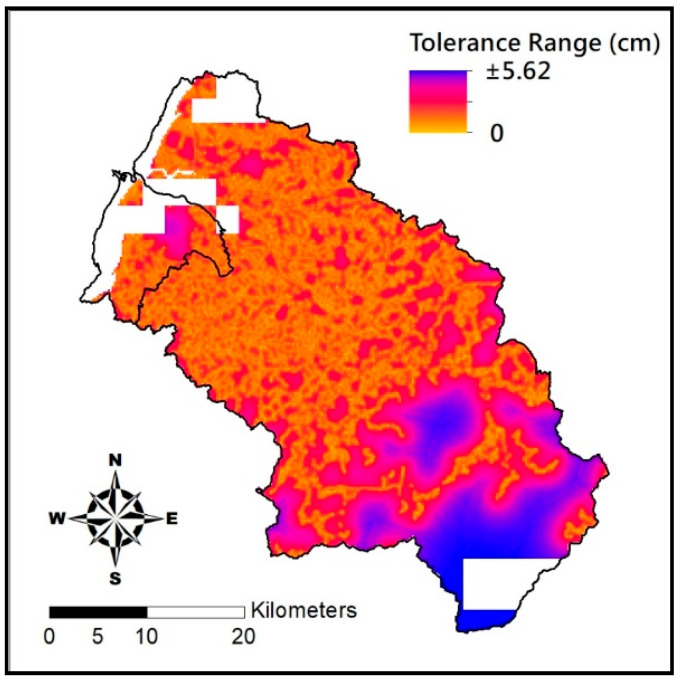
Distribution of soil depth estimation error by BME.

**Figure 10:**
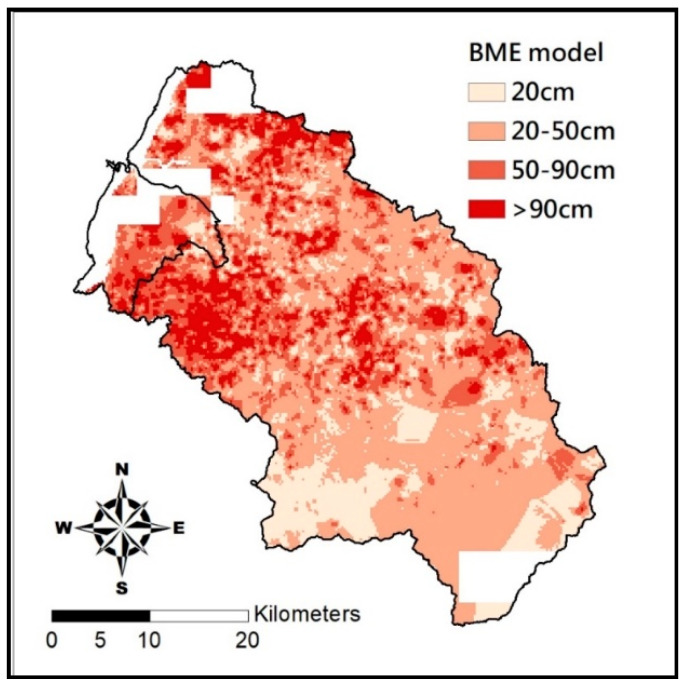
Estimation of soil depth by BME.

The BME method and physiographic factor data of the target points combined may be used to estimate the effective soil depth estimation and the variation and range of the error. At the 95% confidence level, the error range is displayed in Equation (16):

(16) $$d＝\mu \pm 1.96\sqrt{Var}$$

where *d* is the error range of soil depth for a given grid point, *μ* is the mean value that is represented by the estimated soil depth for a given grid point, and *Var* is the variance for a given point. Distribution of the error range of grid points in the Hsinchu district is shown in Figure 9, which suggests that the greater the distances from the estimate point to the actual measurement, the greater the error range will be. As the maximum error range of soil depth estimated by BME in Hsinchu district is a mere 5.62 cm, it is acceptable for existing regulations in terms of soil depth grading. Figure 10 is the soil depth distribution of 5 m grid beads on the proposed BME. With a satisfactory accuracy rate, this map may serve as the reference for soil depth data acquisition in Hsinchu, Taiwan in the future.

### 3.4. Comparison of Estimation Models

With an accuracy rate up to 82.94%, the BME method outperforms the Kriging method in soil depth estimation. In spite of taking data space distribution into account, the Kriging method failed to contain multiple physiographic factors into the analysis. In addition, the Kriging method assumes the actual data follow normal distribution. In the case of non-normal or non-linear space distribution pattern of actual data, it suffers estimation error. The BME method can contain normal and non-normal data and combine hard data and soft data (the soil depth estimation model based on physiographic factor) to obtain estimation. The proposed BME method was applied to the Hsinchu district. Based on the obtained promising results, BME is reported as a useful method that can show the depth of the soil in spatial distribution.

## 4. Conclusions

An appropriate soil depth estimation model is necessary in Taiwan for two reasons: existing soil depth distribution maps are out of date, while the supplemental measurement data available now fails to cover the entire country. This study establishes a soil depth estimation model based on the BME method with input of actual soil depth measurement, physiographic factors, and the selected machine learning model. The BME model was then compared with the traditional Kriging method. The results and conclusions of this study are as follows:

1. The Kriging method is commonly used for space estimation. It suffers in two aspects: first, the statistical assumption of normally distributed estimation data may be not the case of actual soil depth data; secondly, the Kriging method focuses on space distribution characteristics and does not take physiographic factors into account. In spite of being better than existing soil depth distribution diagrams available now in Taiwan, its accuracy rate is a mere 40%.
2. The BME method incorporates both hard data of soil depth measurement and physiographic factor (soft data)-based soil depth estimations to take both natural environmental factors and space distribution characteristics into account at the same time. The BME method not only performs without the normal distribution assumption, but also comes out with much better estimation (80%) than that of the Kriging method.
3. The soil depth distribution map of Hsinchu district in Taiwan produced by the BME method in this study gives soil depth estimation at grid points with an error range of a mere ±5.62 cm. This is acceptable for current soil depth grading standards and may be adopted for districts without soil depth data. This is of great help for disaster prevention and land management of slopeland in Taiwan.

## Figures and Tables

**Table 1:** Soil depth grading standards by the Taiwan Slopeland Conservation and Utilization Act.

Soil Depth Grade	Ratings
Very deep	>90 cm
Deep	50–90 cm
Shallow	20–50 cm
Very shallow	<20 cm

**Table 2:** Correlation between various factors and soil depth.

Physiographic Factor	Correlation with Soil Depth	Distribution Diagram
Slope	Steep slopes tend to have thinner soils which are hard to retain, while gradual slopes may have more pedogenesis and pileup	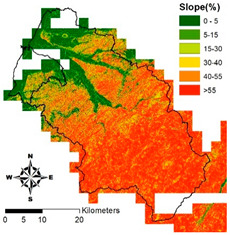
Aspect	Different aspects may feature large differences in solar radiation, water evaporation, and soil moisture. For example, a sunny slope may have greater solar radiation and water evaporation and thinner soil due to more erosion by wind and rainfall than the sunless side	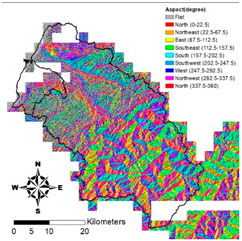
Plan curvature	Perpendicular to the direction of the maximum slope which is related to the convergence and dispersion of water flowing through the surface	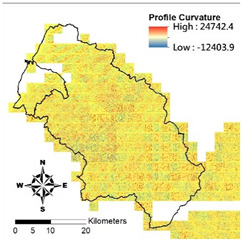
Profile curvature	Parallel to the direction of the maximum slope, that is related to acceleration and deceleration of the water flowing through ground surface and also erosion and accumulation of soil on the slope	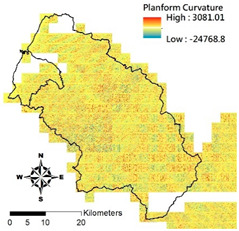
Topographic wetness index	A function of both the slope and the upstream contributing area per unit width orthogonal to the flow direction	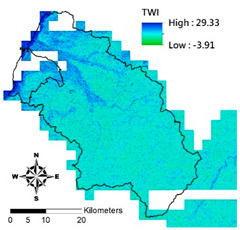

**Table 3:** Soil depth estimation confusion matrix (bold number: correct prediction).

		Soil Depth Measured (cm)			
		<20	20–50	50–90	>90
Soil Depth Estimated (cm)	<20	**(1)**	(2)	(3)	(4)
	20–50	(5)	**(6)**	(7)	(8)
	50–90	(9)	(10)	**(11)**	(12)
	>90	(13)	(14)	(15)	**(16)**

**Table 4:** Soil depth grading by measurements and slopeland soil map (bold number: correct prediction).

		Soil Depth Measured (cm)			
		<20	20–50	50–90	>90
Soil Depth Estimated by Soil Depth Map (cm)	<20	**586**	199	111	11
	20–50	500	**233**	183	16
	50–90	808	473	**461**	91
	>90	426	276	320	**74**
	Accuracy = 28.4%				

**Table 5:** Estimates by the Kriging method on three semivariogram models (bold number: correct prediction).

			Soil Depth Measured (cm)			
			<20	20–50	50–90	>90
Soil Depth Estimated by the Kriging (cm)	Exponential model	<20	**280**	54	1	0
		20–50	1450	**1807**	469	174
		50–90	481	1151	**811**	789
		>90	42	143	143	**422**
		Accuracy = 40.4%				
	Spherical model	<20	**273**	49	1	
		20–50	1449	**1804**	477	171
		50–90	490	1162	**804**	805
		>90	41	140	142	**409**
		Accuracy = 40.04%				
	Gaussian model	<20	**270**	50	1	
		20–50	1451	**1792**	485	177
		50–90	493	1176	**796**	801
		>90	39	137	142	**407**
		Accuracy = 39.74%				

**Table 6:** Accuracy of individual models.

Model	R^2^	MAPE (%)	Hitting Rate
LSSVM	0.12	98.5	0.30
SVR	0.01	70.6	0.37
KNN	0.30	74.9	0.43
LSSVM+KNN	0.11	94.8	0.31
SVR+KNN	0.27	63.7	0.44

**Table 7:** Estimation outcomes of the Bayesian maximum entropy (BME) model (bold number: correct prediction).

		Soil Depth Measured (cm)			
		<20	20–50	50–90	>90
Soil Depth Estimated by BME (cm)	<20	**1632**	143	11	
	20–50	618	**2651**	80	3
	50–90	3	359	**1201**	51
	>90		2	132	**1331**
	Accuracy = 82.94%				
